# Analysis of an AI-powered system for vaccination screening, monitoring, and management in adults aged 50 and above

**DOI:** 10.3389/fpubh.2026.1752720

**Published:** 2026-06-09

**Authors:** Lili Tao, Jiao Zhang, Yunhua Bai, Shuming Li, Bin Jia, Jianxin Ma, Zhi Qi, Zhicheng Yang, Peng Wang, Xiaofeng Wang, Zhenghuan Zheng, Yanling Qiao

**Affiliations:** 1 Beijing Chaoyang District Center for Disease Control and Prevention (Beijing Chaoyang District Health Supervision Institute), Beijing, China; 2 Liulitun Community Health Service Center of Chaoyang District, Beijing, China; 3 Balizhuang Second Community Health Service Center of Chaoyang District, Beijing, China; 4 Wangjing Community Health Service Center of Chaoyang District, Beijing, China; 5 Hujialou Second Community Health Service Center of Chaoyang District, Beijing, China; 6 Hepingjie Community Health Service Center of Chaoyang District, Beijing, China

**Keywords:** AEFI, AI, monitoring, sensitivity, vaccination screening

## Abstract

**Introduction:**

Currently, there is insufficient research on screening contraindications and post-vaccination monitoring for older adults prior to vaccination.

**Methods:**

This study pioneers the integration of artificial intelligence technology, developing a mobile-based medical system (Medduo) that leverages AI for the screening and monitoring of vaccinations in Older adults with chronic diseases. The system was piloted at five vaccination centers. It first assessed residents’ health status and used AI algorithms to recommend appropriate vaccines based on their responses to programmed questions. Personalized suggestions were delivered through mobile terminals. The study compared suspected adverse reactions monitoring by age and gender.

**Results:**

The study data were derived from 2,609 individuals aged 50 and above, of whom 2,599 completed pre-vaccination health screening via mobile terminals. The participants had high rates of previous COVID-19 and influenza vaccinations, at 80.68 and 91.30%, respectively, and 23-valent pneumococcal vaccine and varicella-zoster vaccine vaccination rates of 33.69 and 10.58%, respectively. Most participants had previously been infected with the novel coronavirus, with an infection rate of 74.93%. Analysis of AEFI reports across various age groups showed that the overall incidence of AEFI was 7.83% (207/2,645, equivalent to 7,826.09 per 100,000 population), with the highest report rate observed among those aged 50–59, reaching statistical significance.

**Discussion:**

This self-developed system effectively screened for contraindications in individuals aged 50 or older through intelligent means, reducing the time cost of traditional pre-vaccination screening, and collected AEFI data through a combination of active and passive monitoring, with high sensitivity, contributing to digital health implementation in immunization programs.

## Introduction

As the aging process accelerates, China is now the country with the largest older population in the world; in 2019, there were 176 million individuals aged 65 and above (1). This age group is particularly susceptible to chronic diseases, with their immune systems and overall resistance diminishing as they grow older (2–6). Research indicates that as people age, the risk of contracting infectious diseases such as influenza, respiratory syncytial virus (RSV) infections, community-acquired pneumonia (CAP), and herpes zoster (HZ) increases (7–14). Scientifically using vaccines and conducting preventive inoculations is the most cost-effective and effective method to control infectious diseases and prevent infectious diseases among the older adults.

Given the high prevalence of chronic diseases among the older population, with 75.8% of those aged 60 and above suffering from at least one chronic condition, and the significant burden of multiple diseases, the older population’s health vulnerability is markedly elevated compared to the general adult population. Therefore, it is crucial to screen for contraindications before vaccination and monitor and manage post-vaccination conditions in the older adults. Currently, vaccination clinics have numerous concerns when assessing whether older patients with chronic diseases are suitable for vaccination. For example, some vaccination doctors tend to recommend postponing vaccination for patients with cardiovascular diseases or chronic obstructive pulmonary disease (COPD). However, delaying vaccination actually increases the risk of contracting infectious diseases such as influenza and pneumonia in this population. Therefore, exploring the use of artificial intelligence to scientifically determine contraindications can not only improve the vaccination rate among older patients with chronic diseases but also enhance the efficiency of vaccination doctors in determining vaccination indications for such populations.

Nevertheless, there is a recognized gap in research focusing on the older population, despite the evident need for more comprehensive studies on their vaccination rates and health outcomes. This study introduces artificial intelligence technology for the first time, establishing an AI-based online platform for screening and monitoring vaccination among the older population. In addition, this study also exploratorily research on AI-assisted active AEFI reporting by older chronic disease vaccine recipients. At present, AEFI monitoring in China is mainly passive monitoring. After AEFI occurs, vaccine recipients need to actively contact vaccination clinics to report, but most vaccine recipients are not familiar with the reporting process. This study introduced mobile terminals, significantly enhancing the reporting awareness of vaccine recipients and the sensitivity of AEFI monitoring. A pilot program was implemented at five vaccination centers, examining the prevalence of chronic diseases and the COVID-19 vaccination status among individuals aged 50 and above, prior to their vaccination. The study compared the differences in monitoring suspected adverse events following immunization (AEFI) based on age and gender, aiming to boost the vaccination rate among the older population and fully leverage the role of vaccines in disease prevention and control.

## Materials and methods

### Ethical approval

The study has been approved by the Life Ethics Committee of The Affiliated Friendship Hospital of Capital Medical University (No. 2022-P2-045-01), which is responsible for the overall coordination and arrangement of this research as the leading institution. All personal information has been deleted and only de-identified data were received for this study.

### Data sources

The data come from the Medduo Older Population Chronic Disease Population Vaccination Screening and Monitoring Management System. It is an online platform developed independently, which mainly includes vaccine publicity and education, pre-vaccination screening, vaccination clinic appointment, follow-up at different time points after vaccination and other functions.

### Inclusion criteria

In Chaoyang District, Beijing, five adult vaccination clinics were selected to operate the Medduo Older Population Chronic Disease Population Vaccination Screening and Monitoring Management System. These clinics are located in Balizhuang Second, Hepingjie, Hujialou Second, Liulitun, and Wangjing Community Health Service Centers. From August 1, 2023 to November 30, 2024, individuals aged ≥50 years who used the Medduo Online Platform at these clinics were included in this study.

### Platform applications

The pre-vaccination screening encompasses the essential background of the study participants, including prevalent chronic conditions such as hypertension, diabetes, coronary heart disease, osteoporosis, and chronic obstructive pulmonary disease; recent experiences with COVID-19, acute illnesses, severe chronic disease exacerbations, or respiratory symptoms; and their vaccination profiles. The platform’s homepage offers an in-depth analysis of the benefits and risks of vaccination and promotes vaccination for infectious diseases among people aged 50 and older. In this study, the vaccination clinic provided four types of vaccines to individuals aged 50 and above who were deemed suitable for vaccination after pre-vaccination screening: influenza vaccine, herpes zoster vaccine, 23-valent pneumococcal vaccine, and COVID-19 vaccine. The Medduo Online Platform sent automatic SMS reminders to vaccinated individuals at 24 h, 5 days, 15 days, 6 weeks, and 3 months post-vaccination, along with a link to an online survey questionnaire asking if any adverse events occurred. Upon receiving a report of an adverse event from any vaccinated individual, the Medduo Online Platform promptly notified the vaccination clinic, and urged its staff to contact the individual to gather detailed information about the reaction. The Medduo Online Platform addressed the needs of people aged 50 and older comprehensively by incorporating diverse input methods, such as voice and handwriting recognition, thereby ensuring convenience and accessibility for this age group.

### AEFI monitoring

Based on the National Surveillance Plan for Adverse Reactions to Vaccination (2022 Edition) (15), the study’s monitoring of adverse events following immunization (AEFI) at various time points post-vaccination encompassed: immediate anaphylactic shock within 24 h, allergic reactions without shock (including urticaria, maculopapular rash, laryngeal edema), toxic shock syndrome, syncope, and other conditions. Whether fever (axillary temperature ≥37.3 °C), angioedema, systemic purulent infections (sepsis, bacteremia, pyemia), redness, swelling, induration, or local purulent infections (local abscess, lymphangitis, lymphadenitis, cellulitis) at the injection site, and other adverse reactions occurred within 5 days; whether measles-like or scarlet fever-like rashes, allergic purpura, Arthus reaction, febrile seizures, epilepsy, polyneuritis, encephalopathy, encephalitis, and meningitis occur within 15 days; whether thrombocytopenic purpura, Guillain-Barré syndrome, and other adverse reactions occurred within 6 weeks; and whether arm neuritis, sterile abscesses at the injection site, and other adverse reactions occurred within 3 months, as detailed in recent studies and reports.

### Statistical analysis

The study collected data on the registration of chronic diseases before vaccination, past vaccination history, past COVID-19 infection status, and AEFI. These data were collected through the Medduo Online Platform. Statistical analyses were conducted using two-tailed tests, with results deemed statistically significant when *p*-values were less than the conventional threshold of 0.05. The software SPSS version 22.0 was used for these analyses.

### AI architecture and decision logic

The intelligent screening module of the Medduo system employs a rule-based decision engine grounded in the National Guidelines for Vaccination Contraindications and expert clinical consensus. The decision framework operates through a multi-layered conditional logic: (1) collection of health status data via programmed questionnaires (chronic disease type, acute symptoms, allergy history, recent infections); (2) rule-matching algorithm that cross-references input variables against a predefined knowledge base of contraindications and precautions; and (3) automated classification into three output categories: “suitable for vaccination,” “recommend postponement,” or “temporarily unsuitable.” This deterministic approach ensures clinical interpretability and regulatory compliance, distinct from black-box machine learning classifiers.

The mobile terminal was developed as a multi-end adaptive microservices system (Frontend: Vue.js, native Android/iOS; Backend: Java Spring Cloud; Database: Oracle 11 g).

## Results

### Main features of the data set

**Figure 1:**
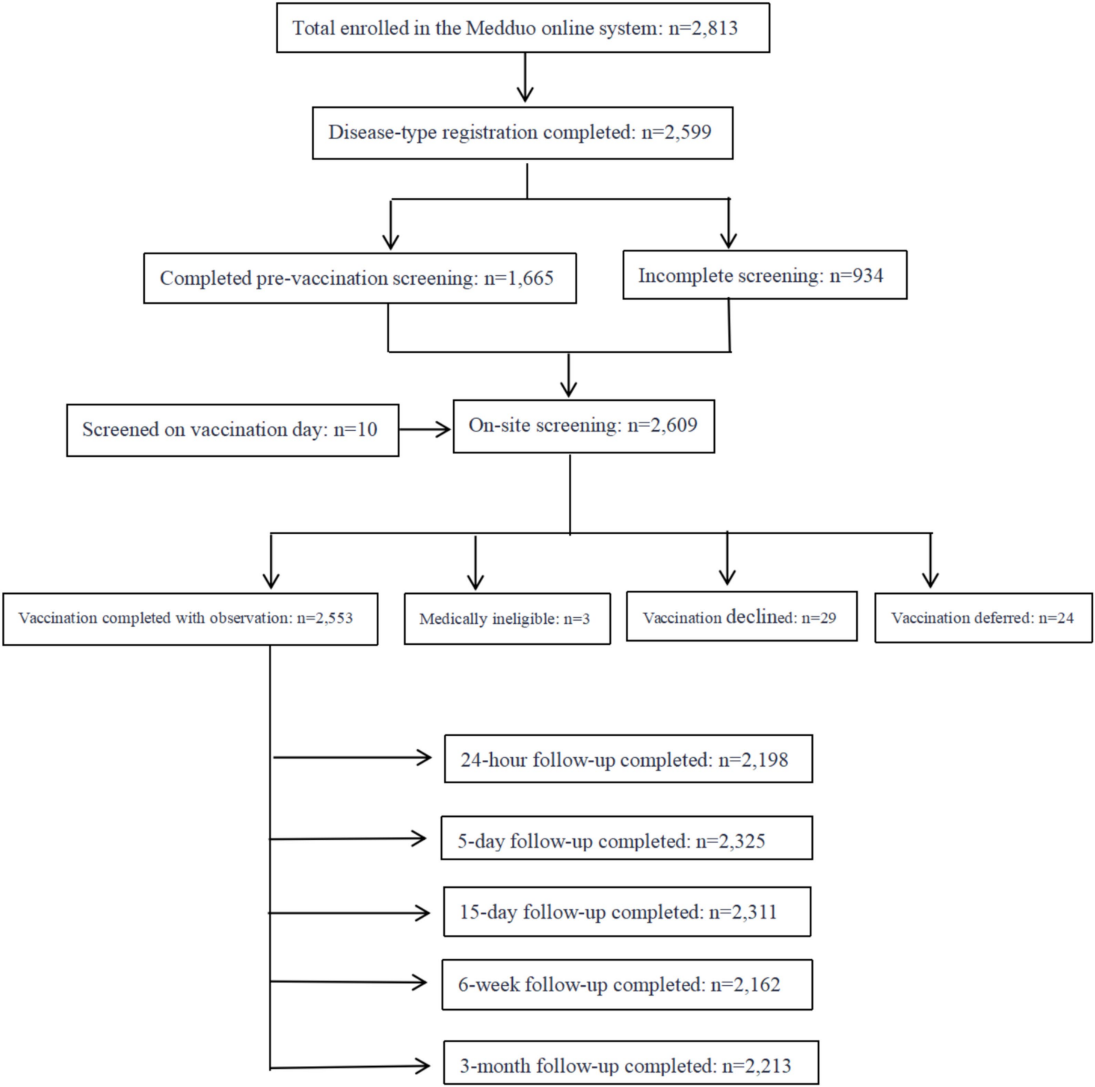
Flow chart of registration and enrollment of people over 50 years old in Medduo platform.

The Medduo Online Platform collected registration records from 2,813 individuals at five adult vaccination clinics in Chaoyang District, Beijing. Among the registrants, 2,599 were registered for vaccination for chronic disease management, and 2,609 individuals successfully completing the on-site screening process. The Medduo Online Platform flagged 24 individuals as potential candidates for vaccination postponement, and another 3 as temporarily ineligible for vaccination. A total of 2,553 individuals underwent on-site vaccinations and were subsequently observed for 30 min. In the follow-up of adverse reactions, 2,198 individuals were followed up for 24 h, 2,325 for 5 days, 2,311 for 15 days, 2,162 for 6 weeks, and 2,213 for 3 months (see Figure 1 and Table 1).

**Table 1:** Chronic disease prevalence of registered personnel in five communities.

**Community**	**Hypertension**		**Diabetes mellitus**		**Coronary disease**		**Osteoporosis**		**Chronic obstructive pulmonary disease**		**Other chronic conditions**		**Number of patients registered (no.)**
	**Number of cases (persons)**	**Proportion (/%)**	**Number of cases (people /%)**	**Proportion (/%)**	**Number of cases (people /%)**	**Proportion (/%)**	**Number of cases (people /%)**	**Proportion (/%)**	**Number of cases**	**Proportion (/%)**	**Number of cases (people /%)**	**Proportion (/%)**	
Bali Zhuang Second	39	9.95	24	6.12	65	16.58	18	4.59	3	0.77	0	0.00	392
Hepingjie	180	37.74	91	19.08	52	10.90	16	3.35	5	1.05	24	5.03	477
Hujialou Second	601	74.38	296	36.63	10	1.24	3	0.37	0	0.00	11	1.36	808
Liulitun	211	37.15	97	17.08	29	5.11	173	30.46	6	1.06	31	5.46	568
Wangjing	152	42.94	72	20.34	47	13.28	43	12.15	3	0.85	19	5.37	354
Total (people /%)	1,183	45.52	580	22.32	203	7.81	253	9.73	17	0.65	85	3.27	2,599

### Screening before vaccination

Through the Medduo online system, pre-vaccination screening was completed for 2,609 individuals. The system identified 24 individuals who should postpone vaccination, 3 individuals who are temporarily not suitable for vaccination, and 29 individuals who had opted out of vaccination. A total of 2,553 individuals completed the recommended vaccination schedule. Analysis of previous vaccination status among the enrolled population revealed variations based on vaccine subsidy levels. The vaccination rate was highest (91.30%) for the fully subsidized COVID-19 vaccine. In contrast, the vaccination rate was lowest (10.58%) for the fully self-paid herpes zoster vaccine, within this age group (details in Table 2). Further analysis of history of novel coronavirus infection within the enrolled population showed that the majority (74.93%) had been previously infected (details in Table 3). Due to the lack of recorded dates for COVID-19 vaccination and infection, these data do not allow for conclusions regarding the protective efficacy of the COVID-19 vaccine.

**Table 2:** Pre-screening of previous vaccination status.

**Name of community**	**Influenza vaccine**		**Herpes zoster vaccine**		**23 percent price of the pneumonia vaccine**		**Novel Coronavirus vaccine**		**Number of people screened before treatment (people)**
	**Number of vaccinations (persons)**	**Proportion (/%)**	**Number of vaccinations (people /%)**	**Proportion (/%)**	**Number of vaccinations (people /%)**	**Proportion (/%)**	**Number of vaccinations (people /%)**	**Proportion (/%)**	
Bali Zhuang Second	372	88.57	20	4.76	224	53.33	381	90.71	420
Hepingjie	394	82.60	99	20.75	170	35.64	443	92.87	477
Hujialou Second	750	93.05	67	8.31	308	38.21	717	88.96	806
Liulitun	299	54.96	68	12.50	106	19.49	510	93.75	544
Wangjing	290	80.11	22	6.08	71	19.61	331	91.44	362
amount to	2,105	80.68	276	10.58	879	33.69	2,382	91.30	2,609

**Table 3:** Statistics of pre-screening of novel coronavirus infection.

**Name of community**	**He has been infected with the novel coronavirus**		**Not infected with novel coronavirus**		**It is not clear whether they have been infected with the novel coronavirus**		**Number of people screened before completion (people)**
	**Number of persons (person)**	**Proportion (/%)**	**(Human being)**	**Proportion (/%)**	**(Human being)**	**Proportion (/%)**	
Bali Zhuang Second	380	90.48	33	7.86	3	0.71	420
Hepingjie	369	77.36	93	19.50	15	3.14	477
Hujialou Second	518	64.27	40	4.96	248	30.77	806
Liulitun	413	75.92	106	19.49	25	4.60	544
Wangjing	275	75.97	51	14.09	36	9.94	362
Amount to	1955	74.93	323	12.38	327	12.53	2,609

### AEFI occurrence

The five community health service centers successfully vaccinated 2,553 individuals on-site and conducted observation for these individuals. During influenza vaccine administration, a strategy was implemented to vaccinate individuals suitable for other vaccines simultaneously with two vaccines. For those aged 50 and older, a total of 2,190 doses of influenza vaccine, 270 doses of herpes zoster vaccine, 174 doses of 23-valent pneumococcal vaccine, and 13 doses of COVID-19 vaccine were administered.

After vaccination, 121 cases of AEFI were reported within 24 h, accounting for 4.74% of the vaccinated population; a total of 207 cases of AEFI were reported over the five follow-up periods, representing 8.11% of the vaccinated population. All 207 cases were categorized as general reactions, including low-grade fever, local redness and swelling, general discomfort, and fatigue. Specifically, 11 individuals experienced redness and swelling at the injection site, 39 experienced pain at the injection site, 25 had a fever, 9 had fatigue, 4 had dizziness or headache, 2 had throat discomfort, and 1 had nausea or cold sweats. No cases of severe reactions, such as high fever (≥38.6 °C), local redness and swelling larger than 2.5 cm, or local hard lumps larger than 2.5 cm, were observed. No abnormal reactions were identified (see Table 4).

**Table 4:** Active monitoring of adverse reactions at different time points after vaccination.

**Vaccination units**	**24 h**			**5 days**			**15 days**			**6 weeks**			**3 months**		
	**Number of adverse reactions (people)**	**Follow-up (number of persons)**	**Adverse reaction incidence (per 100,000)**	**Number of adverse reactions (people)**	**Follow-up (no.)**	**Adverse reaction incidence (per 100,000)**	**Number of adverse reactions (people)**	**Follow-up (number of persons)**	**Adverse reaction incidence (per 100,000)**	**Number of adverse reactions (people)**	**Follow-up (no.)**	**Adverse reaction incidence (per 100,000)**	**Number of adverse reactions (people)**	**Follow-up (no.)**	**Adverse reaction incidence (per 100,000)**
Bali Zhuang Second	0	429	0.00	1	432	231.48	8	432	1851.85	8	431	1856.15	0	421	0.00
Hepingjie	48	461	10412.15	10	476	2100.84	1	477	209.64	0	464	0.00	0	453	0.00
Hujialou Second	12	665	1804.51	4	633	631.91	2	735	272.11	1	731	136.80	2	507	394.48
Liulitun	59	441	13378.68	18	480	3750.00	7	304	2302.63	4	158	2531.65	2	456	438.60
Wangjing	2	202	990.10	5	304	1644.74	12	363	3305.79	0	378	0.00	1	376	265.96
amount to	121	2,198	5505.00	38	2,325	1634.41	30	2,311	1298.14	13	2,162	601.30	5	2,213	225.94

An analysis of AEFI reports across different age groups revealed that the overall incidence of AEFI reports was 7.83% (207/2,645, or 7,826.09 per 10,000). The highest AEFI reporting rate in this study was observed in individuals aged 50–59, while the lowest was in those aged 70–79. The differences in AEFI reporting rates among age groups were statistically significant (see Table 5). No statistically significant difference was found in the differences in AEFI report rates between sexes (see Table 6). The AEFI reporting rates were 3,615.56 per 100,000 population for the influenza vaccine, 4,4943.82 per 100,000 population for the herpes zoster vaccine, and 8,988.76 per 100,000 populations for the 23-valent pneumococcal vaccine. All these rates were higher than those for the same vaccines monitored by conventional methods during the same period at the same vaccination clinics, except for the COVID-19 vaccine, for which a lower number of vaccinated individuals (see Table 7 for details).

**Table 5:** Analysis of AEFI reports by age group.

**Age**	**Number of doses administered**	**Report the number of AEFI cases**	**Report rate (%)**	**χ2**	***p***
50–59 years old	355	68	19.15	78.450	<0.001
60–69 years	1,203	88	7.32		
70–79 years	828	38	4.59		
≥ 80 years of age	259	13	5.02		
Amount to	2,645	207	7.83		

**Table 6:** Analysis of AEFI reporting by sex.

**Sex**	**Number of doses administered**	**Report the number of AEFI cases**	**Report rate (%)**	**χ2**	**p**
Man	1,172	85	7.25	0.327	0.344
Woman	1,473	122	8.28		
Amount to	2,645	207	7.83		

**Table 7:** AEFI reporting on the Medduo online system Versus conventional surveillance.

**Type of vaccine**	**The Medduo online system**			**Conventional Surveillance**		
	**Number of vaccinations**	**Total AEFI reports**	**Overall AEFI incidence (per 100,000)**	**Number of vaccinations**	**Total AEFI reports**	**Overall AEFI incidence (per 100,000)**
Influenza vaccine	2,185	79	3615.56	29,767	8	26.88
Herpes zoster vaccine	267	120	44943.82	3,168	12	378.79
23-day-old pneumonia vaccine	89	8	8988.76	4,070	1	24.57
Novel Coronavirus vaccine	12	0	0.00	981	3	305.81
Amount to	2,553	207	8108.11	37,986	24	63.18

## Discussion

Currently, the monitoring of adverse events following immunization (AEFI) in China is primarily conducted through the AEFI monitoring module of the immunization planning subsystem of the China Disease Prevention and Control Information System. This passive reporting system is the most commonly used and cost-effective method for continuous safety monitoring and evaluation after market launch. However, this method suffers from low sensitivity and potential underreporting. Some domestic scholars have explored active monitoring (16, 17), primarily through face-to-face interviews and telephone follow-ups, to gather and verify information on AEFI occurrences. Unlike traditional passive monitoring, this method heavily relies on human resources and time, which poses potential limitations. Foreign scholars have also investigated active monitoring (18, 19). Integrating these two monitoring methods into a unified platform could harness their respective strengths. Thereby creating a more comprehensive and objective monitoring model. This study actively explored this approach, for the first time using intelligent means, by employing an information-based monitoring platform based on mobile terminals to monitor adverse reactions via a combined active-passive approach.

These detailed data on adverse reactions following COVID-19 vaccination, with a reported rate of 11.86 per 100,000 doses in China’s national surveillance system from December 15, 2020 to April 30, 2021 (20), underscore the importance of enhancing monitoring systems for suspected adverse reactions. Previous studies have indicated that thrombosis is one of the most severe and atypical adverse effects of COVID-19 vaccines (21). Therefore, the AI-assisted active AEFI monitoring system developed in this study is particularly necessary, as it enhances the identification of rare but severe adverse reactions that require immediate medical intervention.

The pre-vaccination disease registration in this study showed that 67.14% of the participants had chronic diseases, primarily hypertension and diabetes. The screening results showed that the vaccination rates for both COVID-19 and influenza vaccines were high among participants aged 50 and older, exceeding 80%. The COVID-19 vaccination rate among individuals aged 60 and older in Beijing closely was consistent with recent surveys, such as the one conducted by Qi Xiaqi et al. (22), which reported a vaccination rate of 80.5%. The influenza vaccination rate was significantly higher than the reported 18.83% for individuals aged 60 and older in Beijing from 2020 to 2021 (23). Furthermore, the vaccination rates for the 23-valent pneumococcal vaccine and the varicella-zoster vaccine among the study participants were significantly higher than the vaccination rates for the pneumococcal vaccine (11.53%) and the varicella-zoster vaccine (0.54%) for people aged 60 and older in Beijing (24) in 2022. This indicates that the study participants had a high level of compliance with previous vaccinations.

The overall incidence of AEFI for the four vaccines in this study was 7.83% (207/2,645, or 7,826.09 per 100,000 cases), which is higher than the overall AEFI incidence rates reported in China, such as the 70.45 per 100,000 cases reported by the National Health Commission as of May 30, 2022, and 11.86 per 100,000 cases reported by the Chinese Center for Disease Control and Prevention from December 15, 2020, to April 30, 2021, indicating that the combined active and passive monitoring approach has good sensitivity. In this study, all 207 AEFI cases were general reactions, representing a higher proportion of general reactions than the rate of AEFI following the inactivated vaccine of enterovirus 71 (EV71) in children as reported by Luo Xiaoyan et al. (25), suggesting that combining active and passive monitoring approach can identify a great number of general reactions. The incidence of AEFI following influenza, herpes zoster, and 23-valent pneumococcal vaccines was all higher than the reported incidence of AEFI for these three vaccines in China, for which AEFI incidence has been monitored from 2016 to 2022, including the period from 2021 to 2022 (26) and the reported incidence of AEFI following the 23-valent pneumococcal vaccine from 2016 to 2020 (27). Compared with conventional passive monitoring at the same institutions during the same period, the Medduo online system demonstrated significantly higher AEFI reporting rates for influenza, herpes zoster, and 23-valent pneumococcal vaccines, indicating a substantial improvement in surveillance efficiency from using an AI-assisted platform for AEFI monitoring. This effect was not observed for the COVID-19 vaccine, likely due to the smaller number of vaccinated individuals in the study population.

This study found no significant difference in AEFI incidence between sexes (Table 6), but there was a statistically significant difference in AEFI incidence across different age groups (Table 5). The 50–59 age group exhibited the highest incidence of AEFI. This may be attributed to the fact that, compared with older individuals, this younger age group is more familiar with the AI monitoring system and tends to promptly report suspected adverse reactions following vaccination. Additionally, compared with those aged over 60, the immune function of individuals aged 50–59 have relatively stronger immune function, thus eliciting a more robust immune response following vaccination. In contrast, the immune function of those aged over 60 gradually declines, resulting in a milder immune response to vaccines, which consequently decreases the likelihood of experiencing short-term adverse reactions.

The primary strength of this study lies in the use of a self-developed AI-assisted screening and monitoring system for older chronic disease patients. This system leverages AI techniques to screen individuals aged 50 and above for contraindications, significantly reducing the time required for conventional pre-vaccination screening. Additionally, the study employed a combined active and passive monitoring approach to collect data on adverse events following vaccination (AEFI), demonstrating highly sensitive. However, one limitation of this study was that participants were mainly recruited through on-site promotions at vaccination clinics, resulting in a limited sample size. Future studies with larger sample sizes are warranted to confirm these findings.

Additionally, it should be acknowledged that the reliance on mobile terminals may introduce a digital divide, potentially excluding older individuals who are not tech-savvy. Although the Medduo system incorporated voice and handwriting recognition to enhance accessibility, and on-site staff provided assistance throughout the registration and follow-up process, the sample may still be biased toward digitally active individuals. This selection bias could skew the AEFI reporting rates, as more tech-comfortable participants might be more proactive in reporting adverse events. Future iterations should consider integrating large-font interfaces, voice navigation, offline reporting options, and community-based assisted reporting to improve inclusivity and reduce this bias.

From a digital health perspective, the Medduo platform exemplifies the WHO SMART Guidelines approach to immunization, which emphasizes standard-based, interoperable digital systems for improving vaccine safety and coverage (28). Recent systematic reviews have shown that digital interventions, including SMS reminders and mobile-based monitoring, significantly enhance vaccination rates in older adults, although with significant heterogeneity across studies (29). The integration of AI-powered screening with active-passive combined surveillance in this study addressed a critical gap identified in current AEFI monitoring systems, where passive surveillance alone often resulted in significant underreporting (30).

## Conclusion and future directions

In conclusion, the combined active and passive medical management system for screening and monitoring older patients with chronic diseases before and after vaccination is more time-efficient than the traditional on-site pre-vaccination screening. It is highly sensitive in collecting information on AEFI occurrences after vaccination and is worthy of wider application. The integration of AI screening with mobile-based active monitoring, including automated SMS reminders for follow-up, aligned with current WHO digital health strategies for immunization and demonstrated a practical solution for enhancing vaccination safety in older populations.

## Data Availability

The original contributions presented in the study are included in the article/supplementary material, further inquiries can be directed to the corresponding author/s.
